# Synthesis of Hydrophobic Propionyl Neohesperidin Ester Using an Immobilied Enzyme and Description of Its Anti-proliferative and Pro-apoptotic Effects on MCF-7 Human Breast Cancer Cells

**DOI:** 10.3389/fbioe.2020.01025

**Published:** 2020-08-31

**Authors:** Na Xia, Wenjing Wan, Siming Zhu, Qiang Liu

**Affiliations:** ^1^School of Food Science and Engineering, South China University of Technology, Guangzhou, China; ^2^College of Life and Geographic Sciences, Kashi University, Kashi, China; ^3^Guangdong Province Key Laboratory for Green Processing of Natural Products and Product Safety, South China University of Technology, Guangzhou, China; ^4^Overseas Expertise Introduction Center for Discipline Innovation of Food Nutrition and Human Health (111 Center), Guangzhou, China

**Keywords:** esterification modification using immobilized enzyme, propionyl neohesperidin ester, lipophilicity of neohesperidin, apoptosis of breast cancer cells, MCF-7

## Abstract

Neohesperidin (NH) is a natural flavonoid glycoside compound with considerable physiological and pharmacological activities. However, its bioavailability is limited due to poor solubility, and few studies have so far attempted improve the solubility and bioavailability of NH. In this study, we structurally modified NH using an immobilized lipase to improve lipophilicity and therefore expand its applicability in lipophilic media as well as enhance its bioavailability *in vivo*. In addition, we aimed investigated the pro-apoptoptotic activity of this new compound (propionyl neohesperidin ester, PNHE) in MCF-7 breast cancer cells using a variety of cellular assays, including the MTT (3-(4, 5-dimethyl- 2-thiazolyl)-2, 5-diphenyl-2-h-tetrazolium bromide assay, assessment of intracellular reactive oxygen species (ROS) levels, and flow cytometry. We successfully synthesized PNHE using immobilized lipases, and the esterification of NH was confirmed by Fourier transform-infrared spectroscopy (FT-IR). Compared to NH, HNPE showed higher anti-proliferative and pro-apoptotic in MCF-7 breast cancer cells, which may be explained by its increased lipophilicity compared to neohesperidin, benefiting to the action of NH on the cancer cell wall. The IC_50_ of PNHE for inducing apoptosis of MCF-7 cells was 185.52 μg/mL. PNHE increased both the proportion of cells in Sub-G1 phase and the cellular ROS content, indicating a certain therapeutic effect of HNPE on breast cancer.

## Introduction

Cancer is a critical global medical issue (Taher et al., 2012), and nearly 13% of deaths each year are caused by cancer (Bashandy et al., 2014). Specifically, lung cancer, breast cancer, colorectal cancer, stomach cancer, and prostate cancer are among main causes of death globally (Taher and Hegazy, 2013). Although there are several effective treatments for cancer, these often still carry many adverse effects including toxicity to normal cells and tissues. Therefore, there is a need to identify safe and natural and safe substances that could be employed as anti-cancer drugs. So far, some natural flavonoids, including hesperidin and naringin, have been reported to have anti-proliferative activity (Dai et al., 2009; Wang et al., 2015). Additionally, natural flavonoid derivatives have also shown potential anticancer properties by inducing tumor apoptosis (Chen and Yu, 2017).

Neohesperidin, a key natural flavonoid glycoside compound derived from the bitter orange (*Citrus aurantium)*, has been found to have potent physiological and pharmacological activities. These effects include a strong anti-inflammatory and neuroprotective activity (Hwang and Yen, 2008), the ability to inhibit differentiation of adult osteocytes (Tan et al., 2016), and an anti-proliferative effect on human liver cancer cells (Bellocco et al., 2009). Additionally, NH is considered to be a potential hypoglycemic agent by adjusting glucose metabolism (Zhang et al., 2012). However, the low solubility of neohesperidin greatly limits its application for therapeutic use. Although bitter substances such as hesperidin can be developed into reactive substances drugs (Uchiyama et al., 2010), there are few reports on the ester derivation and attempted bioavailability improvement of neohesperidin. In order to overcome the poor solubility of flavonoids, these substances are often chemically modified (Sakao et al., 2009; Yang et al., 2012; Yang et al., 2013), but a loss of the phenolic hydroxyl group in the active group of flavonoids caused by this chemical modification may reduce their activity. Simultaneously, chemical modification of flavonoids often causes production of a large number of by-products, which makes separation of target products difficult. The enzyme biocatalysis method is characterized by simple steps, mild reaction conditions, and high product purity, and is therefore commonly used to replace chemical methods (Le Joubioux et al., 2013). Due to the high recognition specificity and efficient conversion to substrates, lipases are often used as bio-catalysts in organic chemical synthesis for esterification (Zagozda and Plenkiewicz, 2008; Sun et al., 2013). Free enzymes have poor stability, which are easily inactivated and difficult to remove. Currently, immobilized enzymes and whole cell catalysis are often used for organic synthesis experiments (Duetz et al., 2001; Zaidan et al., 2012). Various studies demonstrated that esterified flavonoids usually reach enhanced physiological activity and higher bioavailability compared to the original flavonoid compound (Kontogianni et al., 2001). Several flavonoids bioreactive substances, such as arbutin, naringin, puerarin, and dihydromyricetin, have previously been structurally modified to enhance their lipophilicity, thus expanding their application in the lipid environment (Kontogianni et al., 2001; Jiang et al., 2017; Li et al., 2018). For instance, acetylated arbutin can inhibit the bioactivity of melanoma cells and tyrosinase in B16 mice significantly more than unmodified arbutin (Jiang et al., 2017); puerarin has obvious antioxidant activity on erythrocyte hemolysis after the acylation catalyzed by the whole cell (Li et al., 2018); and the procyanidine of esterified grape seeds has anti-proliferative and pro-apoptotic effects on PC3 prostate cancer cells (Chen and Yu, 2017). Enhanced lipophilicity enables these compounds to enter the lipid bilayer of the cell membrane more easily, thus leading to higher bioavailability *in vivo* and greater liposome-based drug delivery potential (Hashimoto et al., 2000).

The aim of this study was to investigate the enzymatic esterification of NH in organic solvents, and to examine the effects of factors such as enzyme source, solvent, reaction temperature, reaction time and substrate concentration on conversion of NH. Additionally, we used several cellular assays, including the MTT assay, flow cytometry, and ROS measurements, to investigate the pro-apoptotic and anti-proliferative activities of the esterification product PNHE on MCF-7 breast cancer cells, thereby providing a theoretical basis for the improvement of NH bioavailability and the development of novel anti-cancer agents.

## Materials and Methods

### Materials

NH (purity ≥ 98%) was donated by Shandong Benyue Biotechnology Co., Ltd. (Dongying, Shangdong, China). We obtained lipase AS, lipaseAK, lipase AYS, Lipozyme CALB, Novozym435, lipase NS, IM-100, Lipozyme RM, and lipaseAK from Novocata Biotechnology Co., Ltd. Fetal bovine serum (BOVOGEN) Dulbecco’s Modified Eagle’s medium (DMEM), pancreatin-EDTA, streptomycin, penicillin, phosphate buffer solution (PBS), and apoptosis detection kits were purchased from BestBio. Sterile cell culture materials, such as syringe filters, 15 mL and 50 mL tubes, 96- pore plates, and pipettes, were purchased from Shanghai Jining Shiye Co., Ltd.

### Enzymatic Esterification of NH

We added 2 mL of organic solvent, containing 0.5 mmol NH, 15 mmol vinyl propionate (VP), and 40 mg/mL lipase, in a 5 mL conical flask, and placed it on a shaker at 180 rpm at 50°C throughout the full reaction, as previously described (Yang et al., 2013). After 24 h, 50 μL of the reaction mixture were removed, diluted 10 times with methanol, and analyzed using high performance liquid chromatography (HPLC). Each experiment was repeated three times. We then investigated the impact of the VP to neoesperidin (VP/NC) molar ratio, lipase content, reaction temperature, and reaction time on the conversion of substrate. After completion of the reaction, the enzyme solution was filtered to terminate the reaction. Next, the solvent was removed by rotary evaporation to obtain the crude products of enzymatic esterification of NH. Finally, the crude products were extracted three times with ice water and ethyl acetate (1:1, v/v), and the ethyl acetate layers were collected and combined. After rotary evaporation and vacuum drying, fat-soluble neohesperidin (PNHE) was obtained.

### Measurement of Neohesperidin Content

We used Wondasil C18 analytical columns (250 × 4.6 mm, 5 μm) and an acetonitrile-formic acid solution mobile phase and performed gradient elution. The UV detector wavelength was 283 nm, and the column temperature of the reverse-phase column was 35°C, the injection volume was 20 μL, and the duration of one gradient program was 30 min. The specific gradient elution program is shown in Table 1.

**TABLE 1:** Gradient elution program for neohesperidin content measurement.

Time (min)	Flow rate (mL/min)	Percent of mobile phase substances (%)	
		A	B
0	1.000	80	20
20	1.000	0	100
25	1.000	0	100
26	1.000	80	20
30	1.000	80	20

*A: 0.1% formic acid solution (in water); B: methanol.*

The conversion of substrate (%) was calculated based on the ratio of the difference between the peak area before and after the neohesperidin reaction and the peak area of the neohesperidin reaction before the reaction for conversion:

$$C\left(\%\right)=\left(A_{0}-A_{i}\right)/A_{0}\times 100\%$$

where A_0_ and A*_*i*_* are peak areas of neohesperidin before and after reaction.

### Purification and Identification of PNHE

The separating and purifying of neohesperidin esterified products were performed using a semi-preparative liquid chromatography containing a chromatographic column Waters C18 (5 μm, 150 × 19 mm) with the following methodology: flow rate: 1.0 mL/min; mobile phase: 0.1% acetic acid aqueous solution and acetonitrile. The detection wavelength was 283 nm. After purification, methanol-*d4* was used as the solvent. The 500 MHz, NMR spec- troscopy (Bruker AV600 Nuclear Magnetic Resonance Spectrometer, Bruker Co., Germany), UHR-TOF-MS (Bruker Co., Germany) and FT-IR (Vector 33, Bruker, Germany) were used to determine the structure of the product.

### Cell Culture

MCF-7 cells were purchased from the Conservation Genetics CAS Shanghai Cell Bank and cultivated in a humidified environment at 37°C with 5% CO_2_ in DMEM medium supplemented with 10% fetal bovine serum, 100 U/mL penicillin and 100 mg/mL streptomycin as previously described (Chen and Yu, 2017).

### Cell Proliferation Analysis

We used the MTT method evaluate the effect of NH and PNHE on the proliferation of MCF-7 cells (Steinhagen et al., 2016). Specifically, MCF-7 cells (1 × 10^4^) were inoculated into 96-well plates, and 100 uL medium containing different dosages of either NH or PNHE were added. After incubating the cells with the compounds for 24 h, we washed them 3 times with PBS and added 200 μL of 0.5 mg/mL MTT to the medium staining for 4 h. We then carefully removed the medium supernatant and added 150 μL of dimethyl sulfoxide to each well. The samples were placed on a shaker at low speed for 10 min to fully dissolve the crystals, and then detected by a full-wavelength microplate reader (OD = 570).

### Apoptosis Analysis and Cell Cycle Analysis by Flow Cytometry

Apoptosis was detected by Annexin V-FITC and PI staining. MCF-7 cells were treated with NH and PNHE (100, 150, and 200 μg/mL) and cultured for 24 h, rinsed with PBS, and then 400 μL of Binding Buffer were added to resuspend cells (to reach a cell density of 1 × 10^6^/mL). Next, 5 μL Annexin V-FITC and 10 μL PI were added in sequence, and then mixed by vortexing and incubated at room temperature for 5 min in the dark. Afterward, we used flow cytometry (CytoFLEX Becam) for detection, and the proportion of apoptotic MCF-7 cells was calculated using Cell Quset software (1 × 104 cells were recorded Each sample). Cell cycle stage was also analyzed by flow cytometry. MCF-7 cells were treated with PNHE (100, 150, and 200 μg/mL) for 24 h, washed 3 times with cold PBS, and fixed overnight in 70% ethanol at −20°C. After another washing step with PBS, MCF-7 cells were incubated in a solution containing 100 μg/mL PI and 100 g μRNase A PBS at 37°C for 30 min. Additionally, the cell cycle stage distribution was analyzed using FlowJo software, and apoptotic cells with hypodiploid DNA content were quantified by assessing the sub-g1 peak in the cell period mode. In each experiment, 1 × 10^4^ cells were recorded in each sample.

### Measurement of ROS Generation

Cells in the logarithmic growth phase were treated with 0.25% trypsin and placed uniformly into a sterile 96-well plate for cell culture. The culture plate was placed in a 5% CO_2_ incubator at 37°C, pre-diluted DCFH-DA was added to each well to a final concentration of 10 μM. The cells were then incubated at 37°C for 30 min in the dark, rinsed with PBS three times, after which different concentrations (100, 150, and 200 μg/mL) PNHE were added to the sample solution in the experimental group, while the control group was treated with an equal volume of DMEM complete medium. After that, the 96-well plate was returned to the CO_2_ incubator for 1 h. With the accumulation of intracellular dichlorofluorescein (DCF) caused by DCFH oxidation, the production of ROS can be assessed by changes in fluorescence intensity. The ROS content of the treated tumor cells was then detected using a fluorescence microplate reader with an excitation wavelength of 488 nm and an absorption wavelength of 525 nm. The tumor cells without PNHE treatment were served as control group.

## Results and Discussion

### Synthesis of PNHE

**FIGURE 1:**
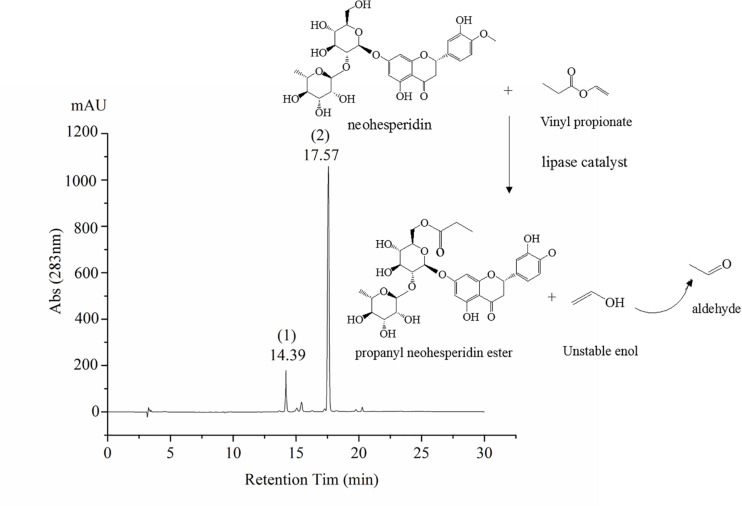
Scheme of PNHE synthesis and high-performance liquid chromatography (HPLC) chromatogram. Peak 1 of HPLC chromatogram is NH, and peak 2 is PNHE.

The synthesis of octyl ferulate, catalyzed by lipases from neohesperidin (0.5 mM) and Vinyl propionate (15 Mm), was performed in an air bath with a total reaction time of 24 h. The PNHE catalyzed by lipases, as well as the liquid samples analyzed by high-performance liquid chromatography (HPLC), are shown in Figure 1.

### Effect of Different Lipases and Solvents on the Conversion of Neohesperidin

**FIGURE 2:**
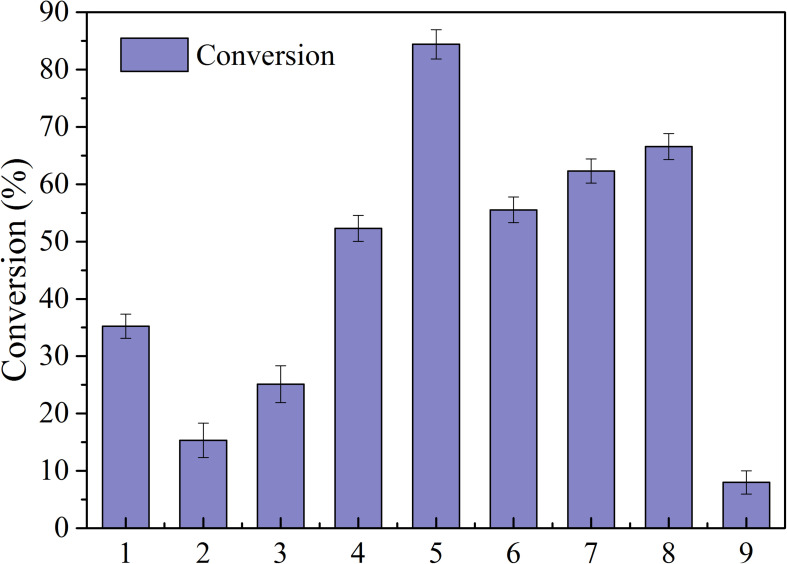
Conversion of NH by different lipases (reaction conditions: NH 0.5mmoL/ml; Vinyl propionate 15 mmoL/mL; enzyme, 40 mg/ml; reaction temperature, 50°C; reaction time, 24 h in t-Pentanol-pyridine (1, lipase AS; 2, lipaseAK; 3, lipase AYS; 4, Lipozyme CALB; 5, Novozym435; 6, lipaseNS; 7, IM-100; 8, Lipozyme RM; 9, lipaseAK).

Lipases are characterized by high activity, mild reaction conditions, and low product requirements, and are commonly used in organic synthesis for food and medicine (Liao et al., 2011). Nine common enzymes (1, lipase AS; 2, lipaseAK; 3, lipase AYS; 4, Lipozyme CALB; 5, Novozym435; 6, lipaseNS; 7, IM-100; 8, Lipozyme RM; 9, lipaseAK) were selected and screened in this study (see Figure 2). The conversion rate using the immobilized lipase Novozym 435 was the highest, reaching 84.42%. Therefore, the immobilized lipase Novozym 435 was selected as the catalyst for the enzymatic esterification of neohesperidin.

Organic solvents play a key role as a reaction medium for ester exchange catalysis of immobilized enzyme. In this study, we investigated nine widely used polar solvents (DMSO, methanol, ethanol, acetone, isopropanol, tetrahydrofuran, tert-butylalcohol, t-Pentanol-pyridine, and isopropyl alcohol) as the reaction medium and results are summarized in Table 2. With t-Pentanol-pyridine, the enzymatic esterification conversion of neohesperidin was the highest. Laane et al. (1987) reported that solvent is closely related to enzyme activity. By comparing the relationship between log p and conversion, it was previously found that lipids prefer hydrophobic solvents, because these solvents may not be able to capture the necessary water for enzymes (Arcos et al., 2001; Romero et al., 2005), thus enhancing stability of biological enzymes. However, polarity may not be the only factor affecting bio-catalyst activity, and the conversion of neohesperidin did not gradually increase with increasing hydrophobicity of the organic solvent. These results further indicated that bio-catalyst activity is not only dependent on the polarity of reactive substances. Each organic solvent has its unique molecular structure, and has unique interference with enzyme molecules. Therefore, bio-catalysts exhibit varying activity (Klibanov, 2001; Li et al., 2018), and the highest conversion medium is considered as the optimal reaction medium.

**TABLE 2:** Effect of Organic Solvents on Acylation of Neohesperidin Catalyzed by Novozym435.

Solvent	logP	Conversion (%)
DMSO	−1.30	52.56 ± 1.11c
Methanol	−0.76	50.28 ± 0.82d
Ethanol	−0.24	54.03 ± 0.71c
Acetone	−0.23	52.53 ± 1.23c
Isopropanol	0.28	44.11 ± 0.65e
Tetrahydrofuran	0.49	65.39 ± 0.24b
tert-butylalcohol	0.70	65.68 ± 0.42b
t-Pentanol-pyridine	0.89	84.34 ± 1.12a
Isopropyl alcohol	1.90	21.43 ± 0.74f

*The reaction time was 24 h. Data are expressed as the mean value (± SD) of three independent experiments; Data marked with a, b, and care significant differences at *p* < 0.05.*

### Effect of Extraction Factors on the Conversion of NH

**FIGURE 3:**
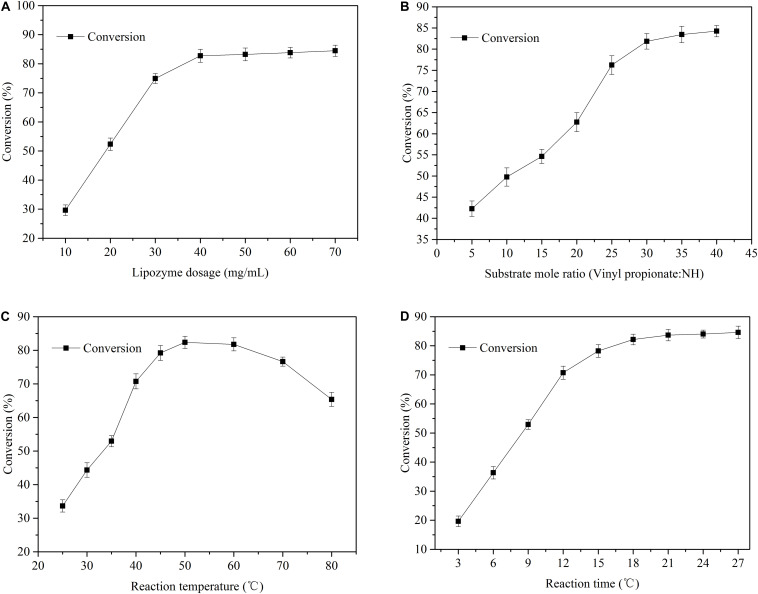
Effects of biocatalyst dosage **(A)**, substrate mass ratio **(B)**, temperature **(C)**, and reaction time **(D)** on the conversion of NH.

Figure 3 shows effects of key factors such as catalyst dosage, reaction temperature, reaction time, and substrate dosage on the esterification of NH. As the catalyst content increased, the conversion of NH increased significantly at an early stage (see Figure 3A). At a catalyst content of 40 mg/mL, the conversion of NH was 82.7%. As the catalyst content increased further, the conversion of NH increased only slightly. Acyl-enzyme complexes were formed by the catalyst at high concentrations, and more active sites were produced. After the catalyst reached saturation, it was affected by substrate concentration and steric hindrance, after which the conversion of substrates could not be further improved, which is consistent with the kinetics of enzymatic reactions (Bidin et al., 2009; Li et al., 2012). As shown in Figure 3B, the molar ratio of substrates had a significant impact on neohesperidin esterification. As the VP/neohesperidin ratio increased from 5:1 to 30:1, the conversion of neohesperidin increased from 42.3 to 81.8%. As the molar ratio continued to increase, the conversion only increased slightly. Specially, when the molar ratio was 40:1, the conversion of the reaction reached 84.2%. Therefore, combining the conversion of the catalytic reaction and the cost of the reaction substrates, the optimal molar ratio was determined to be 30:1.

Reaction temperature has a direct effect on bioactivity and thermal stability of enzymes (Gao et al., 2006). When the reaction temperature is lower than the optimal value of the catalyst, the enzyme activity was inhibited, while when the reaction temperature is higher than the optimal value, the structure of the enzyme can be destroyed or it may lose its activity (Gumel et al., 2013; Sun et al., 2014; Chen and Yu, 2018). Figure 3C investigated the change curve of neohesperidin esterification with temperatures between 25 and 80°C. Specifically, below 50°C, neohesperidin conversion increased continuously and reached a maximum of 82.3% at 50°C. When the temperature was above 50°C, the conversion of substrate was significantly reduced, which is probably due to the denaturation of the enzyme at high temperatures, leading to the inactivation. Figure 3D shows the effects of reaction time on conversion of enzymatic esterification of neohesperidin, where the conversion increased rapidly with the extension of reaction time. Notably, when the reaction reached 18 h, the 82.1% neohesperidin was converted to neohesperidin ester. Then, as time increased, the conversion of neohesperidin slowed down, indicating that neohesperidin reached thermodynamic equilibrium at about 18 h under the catalysis of Novozym435 (Chen and Yu, 2018).

### Identification of the PNHE

**FIGURE 4:**
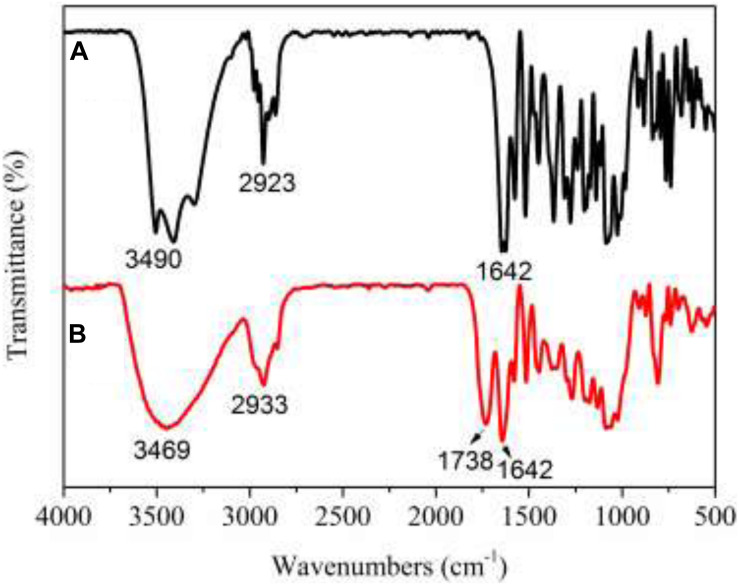
FT-IR spectra of NH **(A)** and PNHE **(B)**.

The FT-IR spectra (see Figure 4) showed a significantly carbonyl (C = O) peak at 1738 cm^–1^ for PNHE which we did not observe for NH. In addition, the intensity of the absorption at 3400 cm^–1^ (O-H stretching vibration) was significantly widened. This was due to the esterification reaction forming a carbonyl group, then a hydrogen bond was formed between the hydroxyl group and the carbonyl group, the group stretching vibration frequency increased, and the absorption band became wider (Thérèse, 1995). These changes confirmed that esterification of NH occurred and that the ester donor replaced the hydrogen atoms of the hydroxyl groups in NH. The PNHE was further identified by means of TOF-MS and 1H NMR spectra. For PNHE, ^1^H NMR (500 MHz, Methanol-*d*_4_) δ 8.00 (s, 1H, -OH_5_), 7.53 (t, *J* = 7.4 Hz, 1H, H_2__’_), 7.42 (t, *J* = 7.7 Hz, 1H, H_6__’_), 6.26 (s, 1H, H_8_), 6.12 (s, 1H, H_6_), 5.57 (s, 1H, H_2_), 5.35 (s, 2H, -OH_3__’_ and -OH_5_), 4.09 (M, 1H, H6” propionyl), 3.35–3.91 (m,8H, H of rhamnoglucosyl),1.28 (m, 3H of CH3 of rhamnosyl), 2.31 (s, 2H, -CH2) 1.10 (s, 3H, -CH3). the molecular ion peak detected showed an m/z at 689 [M + Na]^+^and 705 [M + K]^+^ (mass of PNHE C_31_H_38_O_16_).

### Effect of NH and PNHE on MCF-7 Human Breast Cell Viability

**FIGURE 5:**
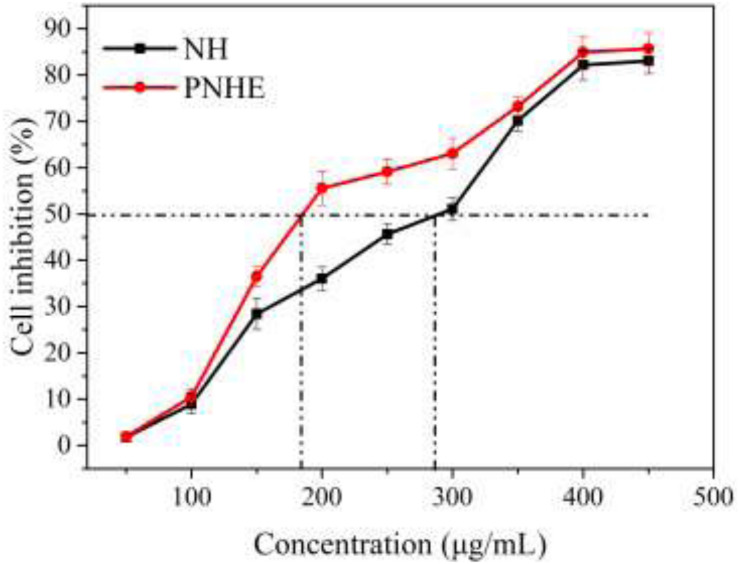
The anti-proliferative effect of NH and PNHE on MCF-7 cells after 24 h treatment. Data are expressed as a percentage of the corresponding control group, and are described as means ± SD (*n* = 6).

The MTT method was employed to explore the anti-proliferative ability of NH and its propionylated derivative on MCF-7 cells, as shown in Figure 5. There was a dose-dependent inhibition on MCF-7 cell proliferation between NH or PNHE concentrations between 50 and 450 μg/ml. After 24 h treatment (50 to 300 μg/ml), PNHE decreased proliferation of MCF-7 cells by 63.1%, while NH only decreased proliferation by 51.1%. The IC_50_ of NH and PNHE on MCF-7 cells were 291.8 ± 2.13 and 185.5 ± 3.22 μg/mL, respectively. PNHE therefore showed an increased anti-proliferative activity compared to NH.

**FIGURE 6:**
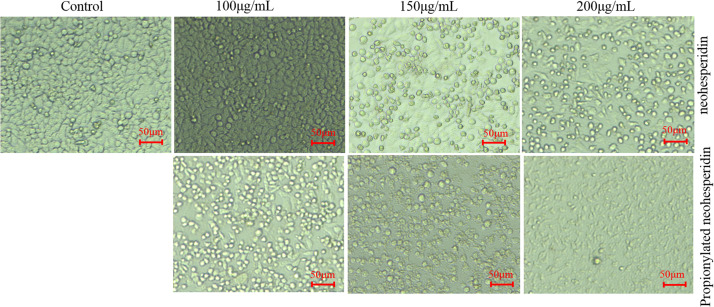
The morphological effect of NH and PNHE on MCF-7 cells. Scale bar = 50 μm. All images shown are representative of three independent experiments.

The morphology of MCF-7 cells treated with different concentrations of NH and PNHE for 24 h is shown in Figure 6. Under normal conditions, MCF-7 cells were polygonal, and the cells were neatly arranged with clear outlines. After treatment with different concentrations of NH and PNHE (100, 150, and 200 μg/mL), we observed strong changes in the cell morphology. With increasing compound concentrations, cells began to shrink and round. Additionally, the number of cells gradually decreased, and ultimately cells lost their unique stretched appearance, showing obvious cytoplasmic vacuolation (Zhang et al., 2015).

### NH and PNHE Induced Apoptosis in MCF-7 Human Breast Cells

Apoptosis is a type of programmed cell death which leads to characteristic morphological changes in cells, including blistering, cell shrinkage, nuclear fragmentation, chromatin concentration, and chromosomal DNA fragmentation (Kim et al., 2015).

**FIGURE 7:**
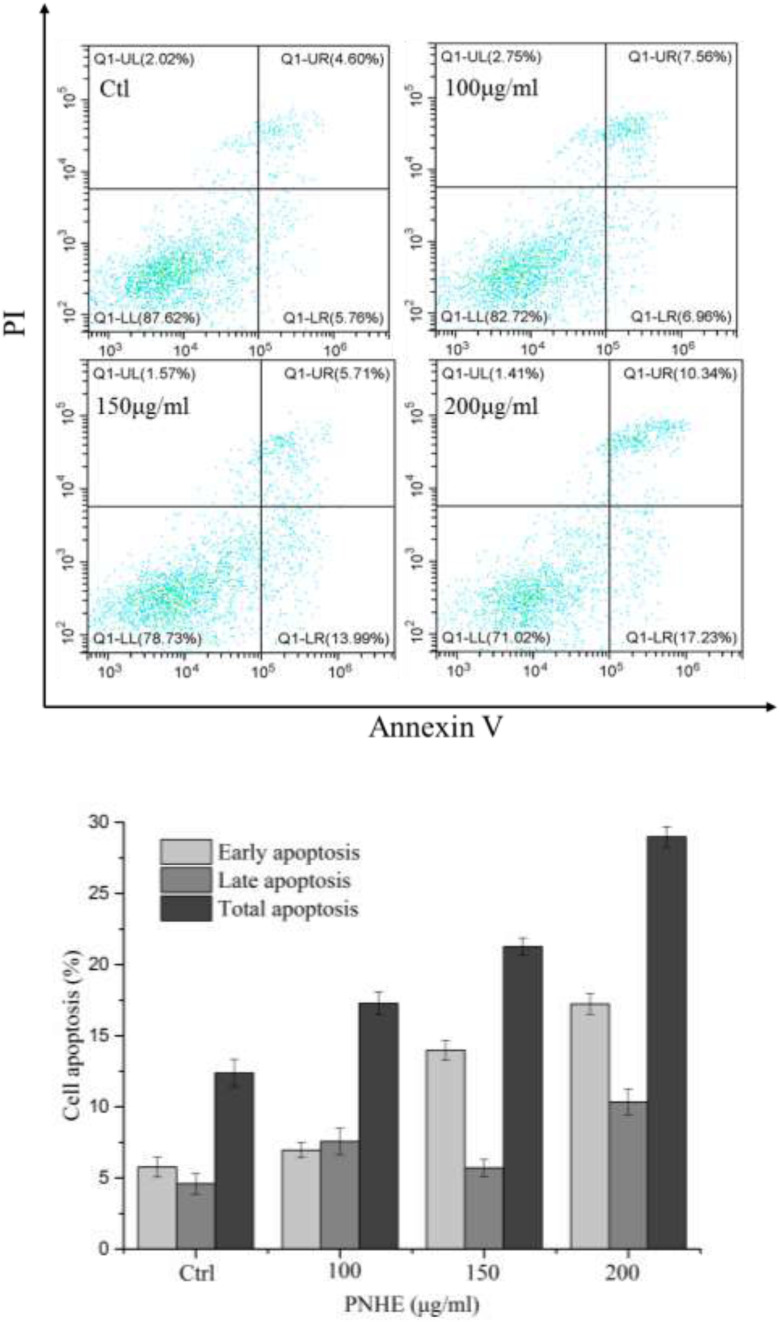
The pro-apoptotic effect of PNHE on MCF-7 cells as observed by double-staining with annexin V and PI and flow cytometry. Data are expressed as a percentage of all cells, and are represented and mean ± standard deviation (*n* = 3).

After treatment with NH and PNHE for 24 h, MCF-7 cells were double-stained with Annexin V-FITC and PI, and quantified using flow cytometry to detect apoptosis. Staining was classified as follows: early apoptotic cells (Annexin V^+^/PI^–^) and late apoptotic cells (Annexin V^+^/PI^+^) (Stojak et al., 2013), and the results are presented in Figure 7. Compared to an apoptosis rate of 3.8% in the control group, the apoptosis rates of MCF-7 cells treated with three different concentrations of NH and PNHE (100, 150, and 200 μg/mL) for 24 h were 4.3, 11, 28.7% and 7.2, 24.3, 77%, respectively.

### Effect of PNHE on the Cell Cycle of MCF-7 Cells

**FIGURE 8:**
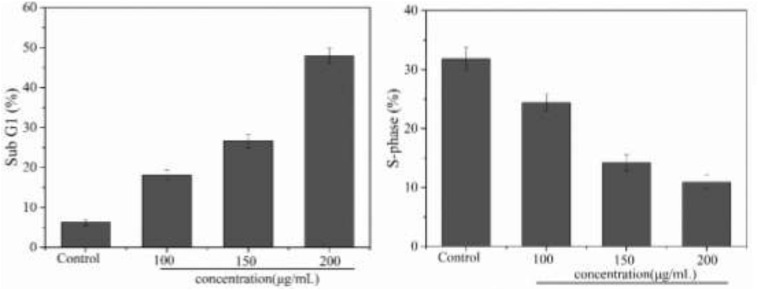
Cell cycle effect of PNHE in MCF-7 cells stained with PI and analyzed by flow cytometry. Sub G1 peak and S-phase content were measured after treatment with different concentrations of PNHE for 24 h. All data are mean ± SD, *n* = 3.

The inhibitory effect of biological samples on the proliferation of cancer cells may be achieved by apoptosis, cell cycle block, or a combination of these two modes (Hirsch et al., 2015). After investigating apoptosis, we therefore also assessed the effect of PNHE on the cell cycle of MCF-7 cells by flow cytometry. MCF-7 cells were treated with PNHE at 10, 150, and 200 μg/mL for 24 h. Following this treatment, we found that the number of cells in the sub-G1 phase showed a significant dose-dependent increase compared to controls, and the number of cells in the sub-G1 phase was 18.1, 26.6, and 47.9%, respectively (Figure 8). Meanwhile, the number of S-phase cells (24.4, 14.2, and 10.9%, respectively), was lower than in the control group (31.8%). The results indicated that cell cycle stasis induced by PNHE plays a role in its growth-inhibitory effect on MCF-7 cells.

### Reactive Oxygen Species (ROS) Levels in MCF-7 Cells Treated With PNHE

**FIGURE 9:**
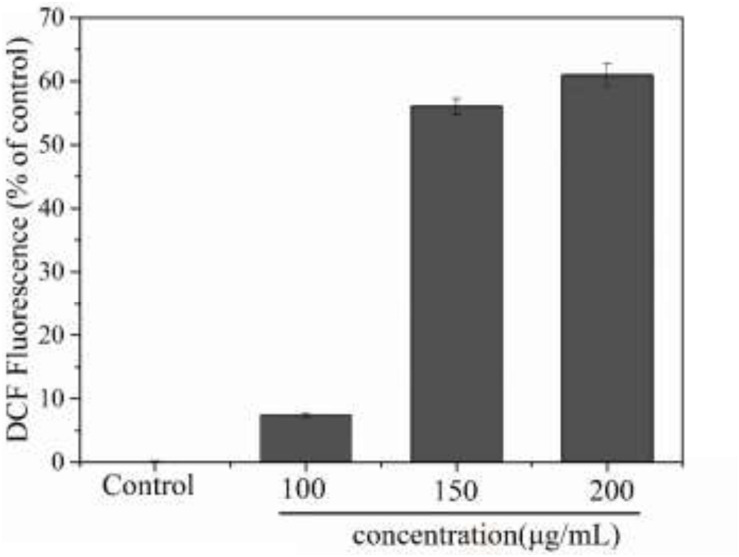
Changes of intracellular ROS level of MCF-7 cells induced by PNHE. Values presented are means ± SD of triplicates.

Reactive oxygen species (ROS) are an important indicator of cellular oxidative stress. Specifically, under normal circumstances, biological systems continuously produce and eliminate ROS, and the antioxidative system in the organism can maintain a metabolic balance. However, when cells are stimulated or stressed by external factors, this system may become dysbalanced. When the ROS content of a cell becomes too high, proteins, nucleic acids, and molecules in the cell membrane may get oxidized, which affects their biological function. Ultimately, this oxidative damage to the cell can inhibit cell proliferation or even result in apoptosis (Saravanan et al., 2003). Additionally, an excessive imbalance of ROS has been shown to be related to many pathological states, including cancer and inflammation, therefore the intracellular ROS content is a significant indicator for evaluating apoptosis (Ghorab et al., 2016). In this experiment, we used the DCFH method to detect ROS in MCF-7 cells. As shown in Figure 9, treatment of MCF-7 cells with PNHE altered the ROS content in cells significantly. At a PNHE concentration of 100, 150, and 200 μg/mL, the ROS content in MCF-7 cells increased to 7.5, 56.1, and 60.9% of controls, respectively, demonstrating a dose-dependent relationship. The results therefore show that PNHE can significantly increase ROS in MCF-7 cells, breaking the metabolic balance of oxygen in cells and inhibiting the proliferation of MCF-7 cells.

## Conclusion

### Resource Identification Initiative

PNHE was synthesized by esterification using NH and VP as substrates under the catalysis of various lipases. When synthesizing PNHE, lipase Novozym435 showed the best catalytic efficiency in tert-butylalcohol. The maximum conversion (82.1%) was reached with a catalyst content of 40 mg/mL, and a VP/NH mass ratio of 30:1 after 18 h of reaction at 50°C. The esterification of NH was confirmed by a change in FT-IR (Figure 4). Additionally, we used the MTT assay to investigate the anti-proliferative effect of NH and PNHE on MCF-7 breast cancer cells. Our results show that both NH and PNHE can significantly inhibit the proliferation of MCF-7 cells in a dose-dependent manner. Compared to NH, PNHE exhibited an improved anti-breast cancer cell line activity. Moreover, we used flow cytometry to detect apoptosis after treatment with PNHE, and our results showed that after 24 h treatment with PNHE, the proportion of apoptotic MCF-7 cells was increased in a dose-dependent manner. We also investigated effects on the cell cycle and found that PNHE treatment of MCF-7 cells for 24 h increased the proportion of cells in the Sub-G1 phase while decreasing the proportion of cells in the S phase. Again, we observed a-concentration dependence in the effectiveness of PNHE treatment in eliciting these changes. Similarly, quantification of ROS confirmed that PNHE has a dose-dependent effect on creation of ROS in MCF-7 cells. To summarize, the above results suggest that PNHE inhibits the growth of MCF-7 cells both by blocking the cell cycle transition from G1 to S and by inducing apoptosis.

This study shows, for the first time, that PNHE has anti-proliferative and pro-apoptotic effects on MCF-7 cells *in vitro*. One of the mechanisms by which PNHE induced an inhibition of growth in this breast cancer cell line was by a block of cell cycle transition, inducing apoptosis. Apoptosis plays a significant role in many biological processes, and together with cell proliferation maintains stability of the internal environment (Renault and Chipuk, 2014; Carrasco-Garcia et al., 2016). In addition, the regulatory barriers of apoptosis are closely related to the development of cancer. Therefore, induction of apoptosis is a key strategy for cancer treatment (Kamal et al., 2014), and PNHE could be used as a potential factor for the prevention and treatment of human breast cancer. Further research will be required to evaluate these findings *in vivo*, such as animal experiments and clinical research, including assessment of safety and efficacy.

## Data Availability Statement

The datasets generated for this study are available on request to the corresponding author.

## Author Contributions

SZ designed and implemented the research. NX and WW performed the experiments and wrote the manuscript. NX supervised the whole process. WW and QL sorted and analyzed the data. All authors contributed in the manuscript revision.

## Conflict of Interest

The authors declare that the research was conducted in the absence of any commercial or financial relationships that could be construed as a potential conflict of interest.
